# Pathogen Identification by Multiplex LightMix Real-Time PCR Assay in Patients with Meningitis and Culture-Negative Cerebrospinal Fluid Specimens

**DOI:** 10.1128/JCM.01492-17

**Published:** 2018-01-24

**Authors:** Karoline Wagner, Burkard Springer, Valeria P. Pires, Peter M. Keller

**Affiliations:** a Institute of Medical Microbiology, University of Zurich, Zurich, Switzerland; b Institute of Medical Microbiology and Hygiene, Austrian Agency for Health and Food Safety, Graz, Austria; Medical College of Wisconsin

**Keywords:** bacterial pathogens, meningitis, molecular detection, multiplex RT-PCR, LightMix kit

## Abstract

Acute bacterial meningitis is a medical emergency, and delays in initiating effective antimicrobial therapy result in increased morbidity and mortality. Culture-based methods, thus far considered the “gold standard” for identifying bacterial microorganisms, require 24 to 48 h to provide a diagnosis. In addition, antimicrobial therapy is often started prior to clinical sample collection, thereby decreasing the probability of confirming the bacterial pathogen by culture-based methods. To enable a fast and accurate detection of the most important bacterial pathogens causing meningitis, namely, Streptococcus pneumoniae, Haemophilus influenzae, Neisseria meningitidis, Streptococcus agalactiae, and Listeria monocytogenes, we evaluated a commercially available multiplex LightMix real-time PCR (RT-PCR) in 220 cerebrospinal fluid (CSF) specimens. The majority of CSF samples were collected by lumbar puncture, but we also included some CSF samples from patients with symptoms of meningitis from the neurology department that were recovered from shunts. CSF samples were analyzed by multiplex RT-PCR enabling a first diagnosis within a few hours after sample arrival at our institute. In contrast, bacterial identification took between 24 and 48 h by culture. Overall, a high agreement of bacterial identification between culture and multiplex RT-PCR was observed (99%). Moreover, multiplex RT-PCR enabled the detection of pathogens, S. pneumoniae (*n* = 2), S. agalactiae (*n* = 1), and N. meningitidis (*n* = 1), in four culture-negative samples. As a complement to classical bacteriological CSF culture, the LightMix RT-PCR assay proved to be valuable by improving the rapidity and accuracy of the diagnosis of bacterial meningitis.

## INTRODUCTION

Bacterial meningitis is the most common and notable infection of the central nervous system, leading to sudden onset of fever, headache, nausea, and altered mental status, and it can rapidly cause death (1). Although the majority of patients with bacterial meningitis survive, neurological sequelae or permanent debilitation persist in as many as one-third of all survivors, especially in newborns and children (2, 3). Predisposing factors like deficiencies of the immune system (e.g., immunosuppressive medication, cancer, diabetes mellitus, alcoholism, human immunodeficiency virus infection) increase the risk of bacterial meningitis (4).

Different bacterial pathogens cause meningitis. However, Streptococcus pneumoniae, Neisseria meningitidis, Streptococcus agalactiae, Haemophilus influenzae, and Listeria monocytogenes were the most prevalent pathogens in bacterial meningitis reported over the last years (5). The prevalence of bacterial pathogens in patients with meningitis also depends on the age of the patient. S. agalactiae is the main cause of neonatal sepsis and meningitis in Western Europe and the United States and an emerging pathogen in immunocompromised adults (6).

N. meningitidis, S. pneumoniae, and H. influenzae are the leading causes of bacterial meningitis worldwide (4, 7–9) with more than 1.2 million cases each year, and neurological sequelae occurring in up to 50% of survivors (10). All three pathogens are carried asymptomatically in the human nasopharynx, and transmission occurs through respiratory droplets or saliva. The introduction of conjugated vaccines (11) reduced the overall incidence of bacterial meningitis. Moreover, it affected the distributions of causative pathogens of bacterial meningitis and the age groups most often affected (4, 11, 12). Another important causative microorganism of meningitis is L. monocytogenes. It is spread by contaminated food, but it is also found in soil, water, and sewage (13, 14). L. monocytogenes has been reported as a common cause of meningitis in young children (15), elderly patients (>60 years), and patients with acquired immunodeficiency (16).

The initial approach to management in a patient with suspected bacterial meningitis includes lumbar puncture (LP) and microbiological examination of the cerebrospinal fluid (CSF) specimen. Empirical treatment with selected third-generation cephalosporins should be initiated as quickly as possible (i.e., ceftriaxone or cefotaxime) after LP (17). However, rapid identification of L. monocytogenes is crucial for optimal outcome, since it is not eradicated by empirical therapy, requiring an additional antibiotic (e.g., ampicillin, amoxicillin, or meropenem) as part of the empirical regimen.

This study was designed as a prospective method evaluation study for rapid identification of bacterial pathogens causing meningitis, namely, S. pneumoniae, H. influenzae, N. meningitidis, S. agalactiae, and L. monocytogenes, using automated DNA extraction (QIAsymphony) from CSF specimens and multiplex real-time PCR (RT-PCR) (<4 h).

## MATERIALS AND METHODS

### CSF specimens and culture.

This study was performed in the diagnostic laboratory at the Department of Medical Microbiology, University of Zurich from January to July 2017. We received cerebrospinal fluid (CSF) samples from patients with meningitis symptoms in sterile screw-cap containers that were collected in secondary and tertiary care hospitals in the Zurich metropolitan area (Switzerland, Europe). In our laboratory routine, first CSF was used for culture, and if there was more than 500 μl leftover CSF, DNA extraction and real-time PCR (RT-PCR) were performed.

CSF specimens were inoculated onto agar plates (5% sheep blood agar, thioglycolate agar, and chocolate agar plates) and into brain heart infusion (BHI) liquid medium. Samples inoculated onto the agar plates were stored in an incubator at 37°C for 24 to 48 h. If growth occurred on the plates, identification of the bacteria was done by matrix-assisted laser desorption ionization–time of flight mass spectrometry (MALDI-TOF MS) (Bruker, Bremen, Germany).

### DNA extraction and multiplex RT-PCR assay.

Five hundred microliters of leftover CSF was stored a maximum of 24 h at 4°C (without freezing and thawing) before DNA extraction and RT-PCR were performed. Prior to DNA extraction, the CSF specimens were spiked with 110 μl of phocine herpesvirus (PhHV) virus (10^5^ virus particles/ml) (European Virus Archive) as extraction and internal RT-PCR amplification control. Virus DNA was coextracted with the clinical samples and coamplified with specific primers in the multiplex RT-PCR. This ensured an accurate control of the whole molecular assay and excluded false-negative results due to inhibition of the PCR. DNA was directly extracted from CSF samples on the QIAsymphony instrument (Qiagen), according to the manufacturer's instructions using the QIA DSP virus/pathogen kit.

The following genetic targets were used in the multiplex LightMix RT-PCR assay. For S. agalactiae detection, the highly specific *cfb* gene, encoding group B streptococcal CAMP factor, was chosen as the LightMix RT-PCR target, as it has shown sensitive and specific detection in previous studies (18). For RT-PCR detection of S. pneumoniae, a specific segment of the pneumococcal autolysin gene (*lytA*) is recommended as the target (19). LytA is a virulence factor involved in autolysis and highly conserved within S. pneumoniae. Furthermore, it has been shown to best separate S. pneumoniae from the genotypically similar species Streptococcus mitis, Streptococcus oralis, and Streptococcus pseudopneumoniae (19). For detection of N. meningitidis, the *ctrA* gene, which encodes an outer membrane protein involved in capsule transport, was used in the LightMix RT-PCR as the target. The *ctrA* gene is highly conserved among the major disease-causing serogroups of N. meningitidis (20). Therefore, it can be used for detection of all encapsulated and most nonencapsulated N. meningitidis isolates (20, 21). H. influenzae RT-PCR detection is based on the protein D gene (*hpd*), which has previously demonstrated excellent sensitivity against a clinically diverse collection of H. influenzae isolates (22). The *hlyA* gene was used as the target in the LightMix RT-PCR, and it has previously demonstrated highly specific and sensitive L. monocytogenes detection (23).

All LightMix primers and probes (TIB Molbiol, Berlin, Germany) used in this study are commercially available and are currently labeled “for research use only.” For RT-PCR, extracted DNA was added to a mixture consisting of PCR-grade water, a LightCycler DNA multiplex master mix (Roche, Rotkreuz, Switzerland), and the LightMix primers and probes (TIB Molbiol, Berlin Germany). RT-PCR master mix composition and the LightCycler (LC) amplification protocol were in accordance with the guidelines provided by TIB Molbiol. The RT-PCR was performed using a LightCycler 480-II (Roche). S. pneumoniae, N. meningitidis, H. influenzae, S. agalactiae, L. monocytogenes, and the PhHV internal control were detected in individual LC channels on the LightCycler 480-II (Roche).

### Retrospective evaluation of the analytical performance of the multiplex RT-PCR.

Culture-negative CSF specimens were pooled and extracted on the QIAsymphony instrument, according to the manufacturer's instructions using the QIA DSP virus/pathogen kit. The analytical sensitivity for each bacterial pathogen was determined by serial dilution of the respective positive-control plasmid in the CSF extracts (5 to 1,000 DNA copies per RT-PCR mixture; see Fig. S1 in the supplemental material). This allowed the generation of a standard curve and the quantification of positive samples. The linearity and measuring range of the RT-PCR were determined for each bacterial pathogen over a range of 3-log-unit dilutions (10 to 1,000 DNA copies per RT-PCR mixture).

To assess the analytical specificity of the multiplex RT-PCR, bacterial strains that are commonly found in clinical specimens or that are part of the human microbial flora were analyzed (see Table S1 in the supplemental material).

Seventeen clinical specimens that have been tested positive by singleplex RT-PCR assay were used for comparative analysis with the LightMix multiplex RT-PCR (Table S2 and Table S3). These singleplex RT-PCRs are routinely used in the reference laboratories in Graz (Austria) and detect *ctrA* (N. meningitidis), *lytA* (S. pneumoniae), *bexA* (H. influenzae), and *hlyA* (L. monocytogenes). Twenty-nine clinical specimens that have been tested positive by PCR amplification of the 16S rRNA genes were analyzed by LightMix multiplex RT-PCR (Table S2 and Table S3). We used 5 μl of DNA that was extracted on the QIAsymphony instrument from clinical specimens and performed PCR amplification of the 16S rRNA genes using the primers, master mix composition guidelines, and the PCR amplification protocol of Bosshard et al. (24). 16S rRNA gene amplification products were purified with the QIAquick PCR purification kit (Qiagen, Hilden, Germany) and sequenced with the forward primer using the BigDye kit and an automated DNA sequencer (ABI PRISM 3130 genetic analyzer; AB Applied Biosystems). SmartGene IDNS software and databases (SmartGene GmbH, Zug, Switzerland) were used for sequence analysis. Homology analysis and species identification were conducted as described previously (24).

### Prospective evaluation of the performance characteristics of the multiplex RT-PCR in comparison to culture.

We evaluated the performance of the multiplex RT-PCR in comparison to culture, which is considered the “gold standard” for the diagnosis of bacterial meningitis, in 220 CSF specimens. In case the two methods showed discrepant results (culture-positive and multiplex RT-PCR-negative test result or vice versa), 5 μl of the extracted DNA from clinical CSF specimens was used for PCR amplification of the 16S rRNA genes and ABI sequencing was performed as described previously (24).

### Statistical methods.

The 2 × 2 contingency table and Cohen's kappa (Κ) were used to calculate agreement between the results of the multiplex RT-PCR and the reference methods (25, 26). All data analysis and data visualization were done in R (27).

### Ethics.

The research was conducted in accordance with the Declaration of Helsinki and national and institutional standards. The act on medical research involving human subjects does not apply to this study. This study was approved by the ethical committee of the canton of Zurich, Switzerland (Req-2017-00605).

## RESULTS

### Retrospective evaluation of the analytical performance of the multiplex RT-PCR.

The analytical sensitivity of the multiplex RT-PCR was evaluated using serial dilutions (1,000 to 5 DNA copies per RT-PCR mixture) of the positive-control plasmids. For all bacterial pathogens analyzed, the limit of detection (LOD) was determined to be at least 10 DNA copies per RT-PCR mixture (2 × 10^3^ DNA copies/ml) (see Fig. S1 in the supplemental material). To determine the analytical specificity of the multiplex RT-PCR, different bacterial isolates were analyzed and tested negative, except one S. pseudopneumoniae isolate that showed a positive RT-PCR amplification result, indicating cross-reactivity with the S. pneumoniae primer/probe set (Table S1).

Moreover, clinical specimens, in which S. pneumoniae, N. meningitidis, H. influenzae, S. agalactiae, and L. monocytogenes were detected by singleplex RT-PCR assays (*n* = 17) or 16S rRNA gene sequencing (*n* = 29), were retrospectively analyzed by multiplex RT-PCR (Table S2 and Table S3). Perfect agreement was found for the detection of N. meningitidis, S. pneumoniae, H. influenzae, and L. monocytogenes in 17 clinical specimens by singleplex RT-PCRs, used in the reference laboratories in Graz, Austria, and multiplex RT-PCR (Table S3). Moreover, comparison of threshold cycle (*C_T_*) values between the singleplex RT-PCRs and multiplex RT-PCR showed lower *C_T_* values in the multiplex RT-PCR for most samples analyzed (Fig. S2). In addition, the results from 16S rRNA gene sequencing and multiplex RT-PCR of 29 clinical specimens agreed completely (Table S2 and Table S3).

### Prospective evaluation of the diagnostic performance of the multiplex RT-PCR in comparison to culture.

In total, 220 CSF samples were analyzed in parallel by culture and multiplex RT-PCR (Table 1). Overall, 16/220 CSF samples were positive by culture. In 10 of these 16 samples, bacteria were found that are not included in the multiplex LightMix RT-PCR panel, namely, Escherichia coli (*n* = 2), Klebsiella pneumoniae (*n* = 1), Serratia marcescens (*n* = 1), Staphylococcus epidermidis (*n* = 4), and Staphylococcus hominis (*n* = 2). All 10 specimens gave negative test results by multiplex RT-PCR (Table 1). In 6/16 CSF samples, S. pneumoniae was detected by culture. Ten of 220 CSF samples revealed positive results by the multiplex RT-PCR. S. pneumoniae was detected in the six culture-positive CSF specimens by multiplex RT-PCR. Additionally, in four culture-negative CSF specimens, N. meningitidis (*n* = 1), S. pneumoniae (*n* = 2), and S. agalactiae (*n* = 1) were detected by multiplex RT-PCR (Table 1 and Table 2). Amplification of the 16S rRNA genes and ABI sequencing detected the same pathogen as identified by multiplex RT-PCR in these four CSF specimens (Table 1). Overall, a high agreement of 99% was found for the detection of bacterial pathogens included in the LightMix RT-PCR panel between culture and multiplex RT-PCR (Table 3).

**TABLE 1:** Detection of bacterial pathogens in CSF (*n* = 220) by multiplex LightMix RT-PCR and culture^*a*^

Bacterium	No. of CSF samples positive for the indicated bacterial pathogen by:		16S rRNA gene sequencing result^*a*^
	Culture	Multiplex LightMix RT-PCR	
H. influenzae	0	0	ND^*b*^
L. monocytogenes	0	0	ND^*b*^
N. meningitidis	0	1	N. meningitidis identified
S. agalactiae	0	1	S. agalactiae identified
S. pneumoniae	6	8	S. pneumoniae identified
E. coli^*c*^	2	0	E. coli identified
K. pneumoniae^*c*^	1	0	K. pneumoniae identified
S. marcescens^*c*^	1	0	S. marcescens identified
S. epidermidis^*c*^	4	0	S. epidermidis identified
S. hominis^*c*^	2	0	S. hominis identified

a 16S rRNA gene sequencing was used to resolve discrepant results.

b ND, not done.

c Not included in the multiplex LightMix RT-PCR panel.

**TABLE 2:** Case study of clinical CSF specimens that were culture negative and tested positive by multiplex LightMix RT-PCR

Case study	Sample	Multiplex RT-PCR result	Microscopic examination result	Clinical feature(s)
70-year-old female	CSF	S. pneumoniae	Cytospin preparations showed mononuclear and polymorphonuclear leukocytes (cell count of >10^3^/ml)	Clinical suspicion of pneumococcal sepsis
			No bacteria were detected by microscopic examination	Patient under antibiotic treatment
39-year-old male	CSF	S. pneumoniae	Cytospin preparations showed mononuclear and polymorphonuclear leukocytes (cell count of >10^3^/ml)	Clinical suspicion of pneumococcal meningitis
			No bacteria were detected by microscopic examination	Patient under antibiotic treatment
58-year-old male	CSF	S. agalactiae	Cytospin preparations showed mononuclear and polymorphonuclear leukocytes (cell count of >10^3^/ml)	Clinical suspicion of meningoencephalitis
			No bacteria were detected by microscopic examination	Patient under antibiotic treatment
26-year-old male	CSF	N. meningitidis	Cytospin preparations showed mononuclear and polymorphonuclear leukocytes (cell count of >10^3^/ml)	Clinical suspicion of meningococcal meningitis
			Gram-negative diplococci were detected by microscopic examination	Patient under antibiotic treatment

**TABLE 3:** Detection of bacterial pathogens in CSF specimens (*n* = 220) by multiplex LightMix RT-PCR and agreement between culture and multiplex RT-PCR results (Κ statistics)

Bacterial pathogen	Multiplex LightMix RT-PCR result	No. of CSF samples with the following culture result:		Κ statistics^*a*^
		Negative	Positive	
H. influenzae	Negative	220	0	Κ = 1
	Positive	0	0	
L. monocytogenes	Negative	220	0	Κ = 1
	Positive	0	0	
N. meningitidis	Negative	219	0	Κ = 0.99
	Positive	1	0	
S. agalactiae	Negative	219	0	Κ = 0.99
	Positive	1	0	
S. pneumoniae	Negative	212	0	Κ = 0.99
	Positive	2	6	

a Κ statistics show the overall agreement between culture and multiplex RT-PCR results.

## DISCUSSION

### This study assesses the performance of a commercial multiplex RT-PCR (LightMix RT-PCR) for the detection of bacterial pathogens causing meningitis.

Retrospective analysis of clinical specimens showed perfect agreement between singleplex RT-PCR, 16S rRNA gene PCR, and multiplex RT-PCR results. The cycle threshold values of the multiplex RT-PCR were similar to those of the singleplex RT-PCR assays, indicating excellent PCR efficacy. In the prospective evaluation, 16/220 CSF samples were culture positive, and 10/220 CSF samples revealed positive results in the multiplex RT-PCR. Moreover, the detection rates of S. pneumoniae, N. meningitidis, and S. agalactiae in CSF specimens were improved by multiplex RT-PCR in comparison to culture. As Switzerland is a country with a low prevalence for bacterial meningitis (28), the low positivity rates observed in this study are reasonable.

Bacterial meningitis is an infectious disease emergency. Prompt diagnosis is essential for targeted antibiotic therapy and optimal outcome (29, 30). Empirically guided meningitis therapy with third-generation cephalosporins is effective against most bacterial pathogens. However, the natural resistance of L. monocytogenes against cephalosporins must be considered (31). Furthermore, rapid identification of N. meningitidis is important in order to promptly administer antimicrobial prophylaxis to close contacts of infected patients (32) and prevent secondary meningococcal meningitis cases. Culture still remains the diagnostic gold standard for detection of pathogens in CSF specimens, which is however handicapped by slow turnaround time (24 to 48 h) and low diagnostic yield when antibiotics were administered to the patient prior to CSF sampling (33). In previous studies, CSF culture was positive in 66% to 88% of patients who were not pretreated with antibiotics, decreasing to a culture positivity rate of 62% to 70% when the patients received antibiotics before lumbar puncture (34, 35). Antibiotic treatment lowers sensitivity of culture which potentially explains why CSF specimens tested negative by culture, but bacterial pathogens were identified by multiplex RT-PCR in four patients included in our prospective study. RT-PCR does not require viable cells, and its diagnostic performance is therefore less affected by antibiotic treatment. Rapid identification of bacterial pathogens by RT-PCR within a few hours after lumbar puncture may help to guide administration of antibiotics and may be especially important if culture yields negative results (17). We propose a rapid diagnostic workflow with low per sample costs (<20 EUR) using an automated DNA extraction device (QIAsymphony) and multiplex LightMix RT-PCR detection. Up to 24 samples can be analyzed in parallel in this workflow within less than 4 h. Therefore, it is suitable for rapid high-throughput routine screening of multiple bacterial pathogens causing meningitis.

There has been much interest in the development of standardized molecular tests for the diagnosis of meningitis (36). Several PCR assays for the simultaneous detection of S. pneumoniae, H. influenzae, and N. meningitidis have been established (20, 37). Also, highly multiplexed assays have been developed that detect viral, fungal, and bacterial pathogens (e.g., BioFire FilmArray meningitis/encephalitis panel; bioMérieux, Marcy l'Etoile, France). However, their application as a first-line test in diagnostics remains controversial (38), as highly multiplexed assays have shown high proportions of false-positive results (36). False-positive CSF test results have the potential to cause significant harm if they lead to the administration of unnecessary, potentially toxic treatment or unwarranted invasive procedures. Alternatively, a negative test result, even when using highly multiplexed assays, does not exclude infection due to organisms that are not included in the panel.

S. pneumoniae was the most frequent false-positively detected organism by the BioFire FilmArray meningitis/encephalitis panel. A suggested explanation for these false-positive results is contamination when CSF specimens were not handled appropriately in the laboratory (36).

By testing 220 CSF specimens, we found high specificity of our multiplex RT-PCR for the detection of bacterial pathogens causing meningitis, as we observed no cross-reactivity of the primer/probe sets. Although the *lytA* gene has been reported to be a very specific target for S. pneumoniae detection, we observed one false-positive amplification signal with an S. pseudopneumoniae culture. This issue has been reported previously (39). There will probably be rare false-positive or false-negative results for virtually any RT-PCR assay for pneumococcal identification, due to recombination events that occur between pneumococci and closely related streptococci and high genomic variability between S. pneumoniae isolates (40). However, the risk of cross-reactivity in CSF is low, because meningitis with other streptococci of the S. mitis group is a rarity without underlying disease (sinusitis, brain abscess).

Our study has several limitations. Our study was designed as a single-center, laboratory-based, prospective method evaluation study. The total number of samples that tested positive for individual organisms in the panel was low in the prospective study, as Switzerland is a country with a low prevalence for meningitis. This did not allow us to calculate the negative and positive predictive values of the LightMix RT-PCR. Furthermore, a negative test result in the multiplex RT-PCR does not exclude infection as a result of organisms that are not included in the panel. Therefore, multiplex RT-PCR will not be able to replace culture but may serve as a valuable complement in routine diagnostics.

In summary, the implementation of new rapid diagnostic tests, like multiplex RT-PCR, as a complement to culture in routine diagnosis of meningitis is crucial. In this prospective study, we evaluated a multiplex RT-PCR that enables rapid identification of the major pathogens of bacterial meningitis, namely, S. pneumoniae, S. agalactiae, H. influenzae, N. meningitidis, and L. monocytogenes in 220 CSF specimens. The multiplex RT-PCR provides a more rapid identification of bacterial pathogens included in the LightMix RT-PCR panel compared to conventional culture. Rapid pathogen identification enables clinicians to adapt the empirical antibiotic regimen, especially if Listeria monocytogenes is detected, and to administer antimicrobial prophylaxis to close contacts of patients with a N. meningitidis infection. This results in better patient management by facilitating targeted therapy and potentially improved clinical outcome.

## Supplementary Material

Supplemental material
