# Supplementary material for: Pathogen Identification by Multiplex LightMix Real-Time PCR Assay in Patients with Meningitis and Culture-Negative Cerebrospinal Fluid Specimens
Source: J Clin Microbiol. 2018 Jan 24;56(2):e01492-17. doi: 10.1128/JCM.01492-17 (PMC5786711; doi:10.1128/JCM.01492-17)
Supplement: Supplemental material [file supp_56_2_e01492-17__index.html]

Supplemental material 

# Pathogen Identification by Multiplex LightMix Real-Time PCR Assay in Patients with Meningitis and Culture-Negative Cerebrospinal Fluid Specimens

## Supplemental material

- Supplemental file 1 -

  Fig. S1 (Evaluation of analytical sensitivity of multiplex RT-PCR by serial dilution of DNA copies from *H. influenzae*, *L. monocytogenes*, *N. meningitidis*, *S. agalactiae*, and *S. pneumoniae*) and S2 (Comparison of *CT* values between singleplex in-house RT-PCR assays and multiplex Lightmix RT-PCR) and Tables S1 (Bacterial isolates used to determine analytical specificity of multiplex Lightmix RT-PCR), S2 (Performance of multiplex Lightmix RT-PCR in comparison to reference methods), and S3 (Performance of multiplex Lightmix RT-PCR in comparison to reference methods)

  PDF, 479K
