# Supplementary material for: Pathogen Identification by Multiplex LightMix Real-Time PCR Assay in Patients with Meningitis and Culture-Negative Cerebrospinal Fluid Specimens
Source: J Clin Microbiol. 2018 Jan 24;56(2):e01492-17. doi: 10.1128/JCM.01492-17 (PMC5786711; doi:10.1128/JCM.01492-17)
Supplement: Supplemental material [file JCM.01492-17_zjm999095826s1.pdf]

Figures.

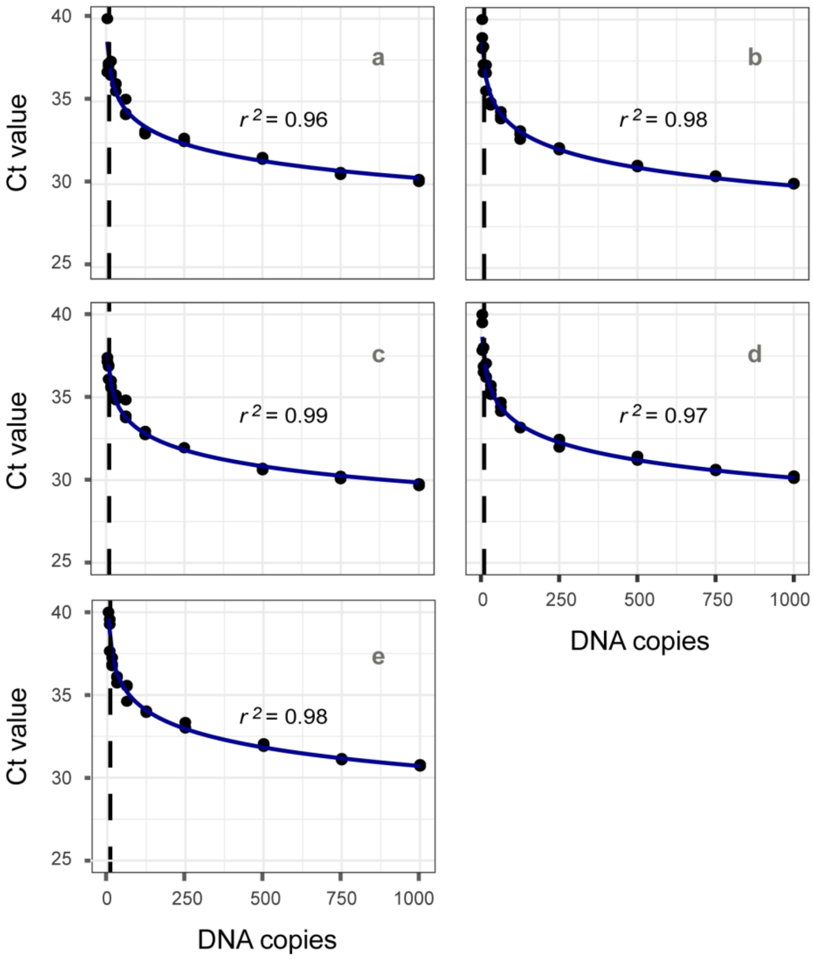

Figure S1. Evaluation of the analytical sensitivity of the multiplex RT-PCR by serial dilution of DNA copies from *H. influenzae* (a), *L. monocytogenes* (b), *N. meningitidis* (c), *S. agalactiae* (d), *S. pneumoniae* (e). Limit of detection at 10 DNA copies per RT-PCR reaction (black dashed line).

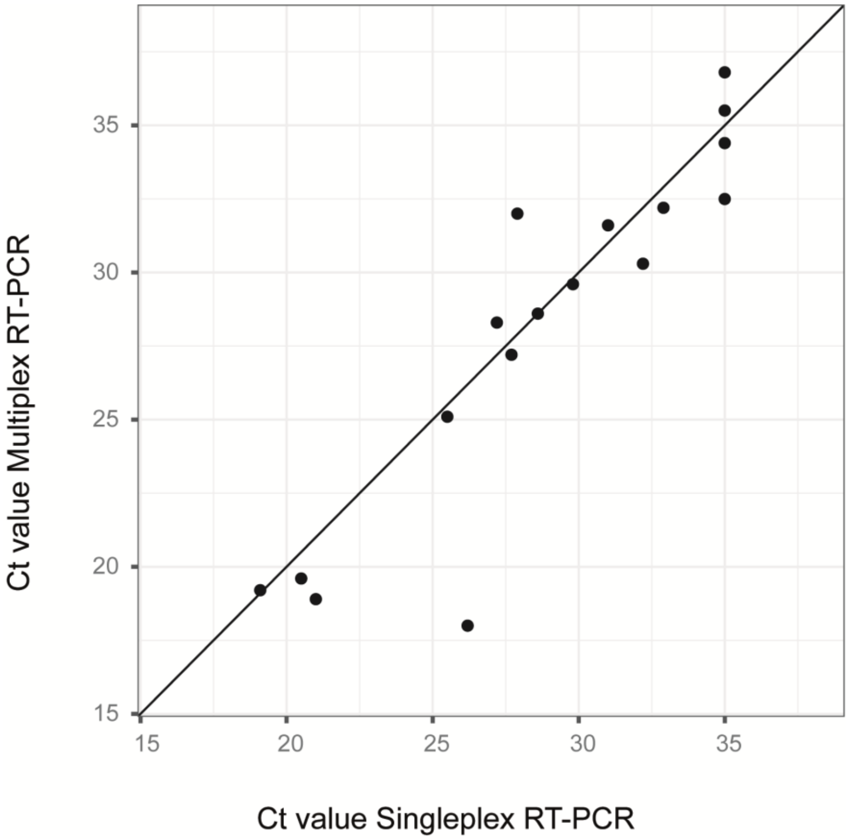

Figure S2. Comparison of Ct-values between singleplex in-house RT-PCR assays and the multiplex Lightmix® RT-PCR.

Tables.

Table S1. Bacterial isolates used to determine the analytical specificity of the multiplex Lightmix® RT-PCR.

	Species	RT-PCR Result
Corynebacterium spp.		
	C. amycolatum	-
	C. propinquum	-
	C. imitans	-
	C. pseudodiphtheriticum	-
Cutibacterium spp.		
	C. acnes	-
Escherichia spp.		
	E. coli	-
Staphylococcus spp.		
	S. aureus	-
	S. caprae	-
	S. epidermidis	-
	S. gallinarum	-
	S. haemolyticus	-
	S. hominis	-
	S. lugdunensis	-
	S. pettenkoferi	-
	S. warneri	-
Streptococcus spp.		
	S. anginosus	-
	S. constellatus	-
	S. bovis	-
	S. gallolyticus	-
	S. gordonii	-
	S. intermedius	-
	S. mitis	-
	S. mutans	-
	S. oralis	-
	S. salivarius	-
	S. pseudopneumoniae	+
Haemophilus spp.		
	H. parainfluenzae	-
Neisseria spp.		
	N. gonorrhoeae	-

Table S2. Performance of the multiplex Lightmix® RT-PCR in comparison to the reference methods (in-house singleplex RT-PCR assays and 16S rDNA sequencing).

Sample ID	Multiplex Lightmix® RT-PCR		Reference method		
	Ct value	Species identification	Method	Result / Ct value	Species identification
264	18.0	*H. influenzae*	Singleplex RT-PCR	26.2	*H. influenzae*
AK557	26.1	*H. influenzae*	16S rDNA sequencing	+	*H. influenzae*
AM599	32.5	*H. influenzae*	16S rDNA sequencing	+	*H. influenzae*
MS543	30.2	*H. influenzae*	16S rDNA sequencing	+	*H. influenzae*
SM138	27.5	*H. influenzae*	16S rDNA sequencing	+	*H. influenzae*
FS292	27.8	*H. influenzae*	16S rDNA sequencing	+	*H. influenzae*
BW383	34.9	*H. influenzae*	16S rDNA sequencing	+	*H. influenzae*
RG485	25.2	*H. influenzae*	16S rDNA sequencing	+	*H. influenzae*
2016128674	29.7	*H. influenzae*	16S rDNA sequencing	+	*H. influenzae*
2016128660	27.0	*H. influenzae*	16S rDNA sequencing	+	*H. influenzae*
234	27.2	*L. monocytogenes*	Singleplex RT-PCR	27.7	*L. monocytogenes*
PCR6	31.6	*L. monocytogenes*	Singleplex RT-PCR	31.0	*L. monocytogenes*
156-07	28.6	*L. monocytogenes*	Singleplex RT-PCR	28.6	*L. monocytogenes*
51-10	29.6	*L. monocytogenes*	Singleplex RT-PCR	29.8	*L. monocytogenes*
2017127059	25.6	*L. monocytogenes*	16S rDNA sequencing	+	*L. monocytogenes*
2017127372	37.2	*L. monocytogenes*	16S rDNA sequencing	+	*L. monocytogenes*
MG8616	30.3	*N. menigitidis*	Singleplex RT-PCR	32.2	*N. menigitidis*
221	28.3	*N. menigitidis*	Singleplex RT-PCR	27.2	*N. menigitidis*
4216	32.5	*N. menigitidis*	Singleplex RT-PCR	35.0	*N. menigitidis*
105	32.2	*N. menigitidis*	Singleplex RT-PCR	32.9	*N. menigitidis*
140	35.5	*N. menigitidis*	Singleplex RT-PCR	35.0	*N. menigitidis*
516	18.9	*N. menigitidis*	Singleplex RT-PCR	21.0	*N. menigitidis*
1316	32.0	*N. menigitidis*	Singleplex RT-PCR	27.9	*N. menigitidis*
2017126185	17.8	*N. menigitidis*	16S rDNA sequencing	+	*N. menigitidis*
2016129092	24.3	*S. agalactiae*	16S rDNA sequencing	+	*S. agalactiae*
2016129431	25.0	*S. agalactiae*	16S rDNA sequencing	+	*S. agalactiae*
162-43	36.8	*S. pneumoniae*	Singleplex RT-PCR	35.0	*S. pneumoniae*
152-26	34.4	*S. pneumoniae*	Singleplex RT-PCR	35.0	*S. pneumoniae*
205-36	19.6	*S. pneumoniae*	Singleplex RT-PCR	20.5	*S. pneumoniae*
127-03	25.1	*S. pneumoniae*	Singleplex RT-PCR	25.5	*S. pneumoniae*
45-11	19.2	*S. pneumoniae*	Singleplex RT-PCR	19.1	*S. pneumoniae*
JO269	33.7	*S. pneumoniae*	16S rDNA sequencing	+	*S. pneumoniae*
MN055	34.1	*S. pneumoniae*	16S rDNA sequencing	+	*S. pneumoniae*
SD547	32.8	*S. pneumoniae*	16S rDNA sequencing	+	*S. pneumoniae*
2016128546	23.1	*S. pneumoniae*	16S rDNA sequencing	+	*S. pneumoniae*
2016128832	24.5	*S. pneumoniae*	16S rDNA sequencing	+	*S. pneumoniae*
2016129497	30.3	*S. pneumoniae*	16S rDNA sequencing	+	*S. pneumoniae*
2016129638	29.6	*S. pneumoniae*	16S rDNA sequencing	+	*S. pneumoniae*
2017126258	36.7	*S. pneumoniae*	16S rDNA sequencing	+	*S. pneumoniae*
2017316085	22.0	*S. pneumoniae*	16S rDNA sequencing	+	*S. pneumoniae*
2017127110	37.8	*S. pneumoniae*	16S rDNA sequencing	+	*S. pneumoniae*
2017127111	32.6	*S. pneumoniae*	16S rDNA sequencing	+	*S. pneumoniae*
2017127222	25.4	*S. pneumoniae*	16S rDNA sequencing	+	*S. pneumoniae*
2017127365	28.0	*S. pneumoniae*	16S rDNA sequencing	+	*S. pneumoniae*
2017127366	26.6	*S. pneumoniae*	16S rDNA sequencing	+	*S. pneumoniae*
2017127535	29.5	*S. pneumoniae*	16S rDNA sequencing	+	*S. pneumoniae*

Table S3. Performance of the multiplex Lightmix® RT-PCR in comparison to the reference methods (in-house singleplex RT-PCR and 16S rDNA sequencing).

			Reference Methods		
H. influenzae	Multiplex Lightmix® RT-PCR		Negative	Positive	Ƙ = 1
		Negative	0	0	
		Positive	0	10	
			Reference Methods		
L. monocytogenes	Multiplex Lightmix® RT-PCR		Negative	Positive	Ƙ = 1
		Negative	0	0	
		Positive	0	6	
			Reference Methods		
N. meningitidis	Multiplex Lightmix® RT-PCR		Negative	Positive	Ƙ = 1
		Negative	0	0	
		Positive	0	8	
			Reference Methods		
S. agalactiae	Multiplex Lightmix® RT-PCR		Negative	Positive	Ƙ = 1
		Negative	0	0	
		Positive	0	2	
			Reference Methods		
S. pneumoniae	Multiplex Lightmix® RT-PCR		Negative	Positive	Ƙ = 1
		Negative	0	0	
		Positive	0	20	
